# Family and population studies by the Adelaide Kuru Team, 1957–1965

**DOI:** 10.1098/rstb.2008.4006

**Published:** 2008-11-27

**Authors:** J.H. Bennett

**Affiliations:** University of AdelaideAdelaide, SA 5000, Australia

A genealogical survey of some members of the Fore population living near Okapa was carried out by Prof. H. N. Robson and Dr F. A. Rhodes in December 1957. This was shortly after the publication of the first paper on kuru in which Zigas & Gajdusek (1957) reported, *inter alia*, the unusual sex and age distribution in victims of the disease (with adult males only rarely affected and female victims apparently comprising two separate classes—childhood and adult) and mentioned the possibility of a genetic predisposition. In the genealogical survey, the incidence of kuru in males was almost equal to that in young females and most of the mothers of these two classes of victims (i.e. males and young females) themselves were said to have died of kuru. This finding and examination of the pedigrees led to the tentative suggestion by Bennett *et al.* (1958) that kuru might be controlled by an autosomal gene **** dominant to its allele **** in females and recessive in males, with the early onset (childhood) cases in females being homozygotes **KK**. These studies were extended in 1959 with the support of the University of Adelaide, the Public Health Department of Papua New Guinea (PNG) and a grant from the Rockefeller Foundation. Essentially the same family patterns were found in all parts of the kuru region that were visited.

When the high frequency of kuru, especially in the South Fore, made it seem increasingly unlikely that the genetic interpretation was tenable, attention was directed to the study of ‘dietary, ritual or medicinal practices which are, or perhaps were, limited almost entirely to adult females and children and only rarely extended to adult males’ (Bennett 1962). In June 1961, two anthropologists, Robert and Shirley Glasse, were recruited as University Research Fellows (with the support of the Rockefeller Foundation grant) to study these and related questions in the kuru region. Their research assignment involved settling in a South Fore village and after becoming fluent in the Fore language learning as much as possible about relevant dietary practices, etc., referred to above. In a letter of 24 October 1961, Bob Glasse wrote, ‘We are now fairly confident that kuru is of recent origin…: it begins to look as if kuru began in the North Fore about 1925 and reached the South Fore within the space of five years. The early incidence was very low… (and) it is only in the past ten or fifteen years that the incidence has increased and that men have begun to fall ill.’ In Adelaide in April 1962 Bob Glasse reported that, until approximately 8 years previously, all corpses in the kuru region were eaten; this cannibalism was practised almost exclusively by the women who fed the semi-cooked flesh to the children and themselves, the daughters generally having the brain and the rest of the head. Berndt (1958) had mentioned cannibalism involving kuru victims in the Fore but R. M. Glasse uncovered the very significant role of women in feeding the semi-cooked flesh to children and themselves. This led to an understanding not only of the characteristic sex and age distribution of kuru victims but also of how the disease had been spread and, later, with the cessation of cannibalism, an explanation for the steady decline in incidence (Glasse 1967).

Among those who deserve to be remembered for their outstanding work caring for kuru victims and their families, special mention should be made of Dr Andrew Gray, who was the first Medical Officer to be appointed to Okapa by the PNG Department of Public Health. For 3 years from early 1959, he not only provided general medical services for the whole area but also took a keen interest in the kuru problem and with the help of two nursing sisters from the Lutheran Mission in New Guinea provided care in the Okapa Hospital for many young orphans whose mothers had died of kuru. The Lutheran Kuru Centre was later established at Awande and in February 1964 when hospital amenities (blankets, baby food and clothing, etc.) from the Australian Red Cross Society were delivered there, it is recorded that 128 orphans and kuru victims were being cared for.
